# Surviving water scarcity: seasonal contrasts in drought and desiccation tolerance of co-occurring *Barbacenia gentianoides* and *Vellozia caruncularis*

**DOI:** 10.3389/fpls.2025.1642013

**Published:** 2025-09-26

**Authors:** Evandro Alves Vieira, Giselly Mota da Silva, Marilia Gaspar, Marcia Regina Braga, Cecilio Frois Caldeira

**Affiliations:** ^1^ Vale Institute of Technology, Belém, Brazil; ^2^ Department of Biodiversity Conservation, Institute of Environmental Research, São Paulo, Brazil

**Keywords:** carbohydrates, metabolic profiling, photoprotection, phytohormone signalling, resurrection plants, Velloziaceae

## Abstract

*Campos rupestres* are tropical highland ecosystems characterized by herbaceous vegetation, high biodiversity, and elevated levels of endemism. Recognized as global biodiversity hotspots, they are increasingly threatened by intense anthropogenic pressures. Plants inhabiting these ecosystems face harsh environmental conditions, including dry winters, intense solar radiation, and shallow, quartzite-derived soils with low water retention capacity. This study examines the differential drought responses of two co-occurring Velloziaceae species, *Barbacenia gentianoides* and *Vellozia caruncularis*, throughout the seasonal cycle under natural field conditions. Ecophysiological and metabolic analyses reveal that *B*. *gentianoides* copes with the dry season by reallocating carbon to the leaf base during senescence, supporting leaf resprouting at the onset of the rainy season. In contrast, *V*. *caruncularis* exhibits desiccation tolerance by preventing senescence in younger leaves during the dry season and maintaining their structural integrity upon rehydration. Distinct metabolic shifts in sugars, amino acids, and secondary metabolites underscore the contrasting strategies of the two species: *V*. *caruncularis* emphasizes osmoprotection and reactive oxygen species (ROS) scavenging, whereas *B*. *gentianoides* focuses on starch and polyol storage for the production of new leaves. Differences in hormone signaling and flavonoid accumulation further underscore species-specific responses, contributing to the regulation of extreme dehydration tolerance in *V*. *caruncularis* and facilitating ethylene-mediated senescence as a survival strategy in *B*. *gentianoides*. Given the limited understanding of drought and desiccation tolerance mechanisms in native rock outcrop species under natural conditions, our findings offer valuable insights into the metabolic adaptations that enable survival in these unique and challenging ecosystems.

## Introduction

*Campos rupestres* are tropical highland landscapes characterized by a distinct phytophysiognomy, consisting of an herbaceous layer interspersed with sclerophyllous shrubs and sub-shrubs, arranged in a mosaic of microhabitats (Colli-Silva et al., 2019; Bugado et al., 2025). Climatically, these ecosystems experience wet summers, dry winters, high vapor pressure deficit (VPD), and elevated total solar radiation. The soils are typically shallow, formed from a matrix of decomposed quartzite rocks with fissures and sand, and contain only minimal organic matter, resulting in low water-holding capacity (Negreiros et al., 2014; Oliveira et al., 2016). *Campos rupestres* are recognized as a global biodiversity hotspot due to their exceptional species richness and high levels of endemism. Despite covering only 0.8% of the country’s territory, they host approximately 15% of Brazil´s vascular flora (Fernandes et al., 2020). However, these ecosystems face escalating threats from intense anthropogenic pressures and projected climate change scenarios in the near future (Arruda et al., 2023; Bugado et al., 2025).

Plants inhabiting these water-limited environments exhibit a range of contrasting water-use strategies that enable them to cope with irregular water availability. These strategies span a continuum from homoiohydric species, which actively regulate water and maintain relative hydration, to poikilohydric or desiccation-tolerant species (Teodoro et al., 2021). The relative water content values at the point of turgor loss observed across species may reflect a threshold of maximal dehydration that most plants can withstand before experiencing irreversible damage or death (Sapes and Sala, 2021). Drought-tolerant species survive under low tissue water content through various physiological strategies (Bartlett et al., 2014). Conversely, desiccation-tolerant plants can survive extremely low tissue water content (≤ 10% WC) and rapidly resume regular metabolic activity upon rehydration (Vieira et al., 2017, 2022, 2024). Carbon acquisition in desiccation-tolerant species is restricted to hydrated and metabolically active tissues. Desiccation-sensitive species, in turn, sustain carbon assimilation during dry periods, albeit with reductions in photosynthesis, stomatal conductance, and water potential (Oliveira et al., 2016). Desiccation-sensitive plants may outcompete smaller, slow-growing resurrection plants in water-limited regions using typical drought-avoidance strategies (Marks et al., 2021). However, they are unable to survive significant water loss (Ntuli, 2012).

Responses to varying degrees of dehydration differ among species and are closely linked to their respective survival strategies. The desiccation-sensitive species *Selaginella moellendorffii* exhibits a limited metabolic response to dehydration stress, marked primarily by slight increases in polyols. In contrast, *S*. *lepidophylla* displays a constitutive desiccation tolerance, relying on a broad spectrum of metabolites and some inducible compounds to withstand water deficit (Yobi et al., 2012). Similar contrasts in morphological and biochemical tolerance strategies have been documented in species of *Eragrostis* (Balsamo et al., 2006) and *Sporobolus* (Oliver et al., 2011). Distinctive traits, including increased leaf tensile strength and the accumulation of sucrose, arginine, asparagine, and antioxidants, often characterize desiccation-tolerant species. In the desiccation-tolerant species *Lindernia brevidens*, gene expression remained relatively stable throughout the transition from severe dehydration to complete desiccation, reflecting physiological resilience. Conversely, the desiccation-sensitive *L. subracemosa* exhibited extensive differential gene expression during this phase, likely as a strategy to delay or avoid the imminent senescence (VanBuren et al., 2018).

The Velloziaceae (Pandanales) is the most prevalent and species-rich vascular plant family in the flora of *campos rupestres*. Its species typically inhabit rocky and shallow-soil environments, particularly on rocky inselbergs in Africa and South America (Porembski and Barthlott, 2000). Predominantly heliophytic, members of this family encompass a wide range of growth forms, including small trees, shrubs, compact rosettes, large stem rosettes, and graminoid-like forms. A diversity of water-related ecological strategies has been proposed as key traits underlying the dominance and distribution of Velloziaceae species in *campos rupestres*. These species are well adapted to seasonally dry areas, particularly those in *Barbacenia* and *Vellozia.* While some species exhibit desiccation tolerance, others are desiccation-sensitive but display notable drought tolerance. In these fields with marked soil and topographic gradients, the small, desiccation‐tolerant Velloziaceae are commonly confined to exposed rock surfaces, whereas larger, less tolerant species exploit deeper soils (Alcantara et al., 2015, 2018).

Comparative systems offer powerful frameworks for uncovering the physiological and metabolic bases of complex biological traits. Variation in water-use strategies under environmentally stressful conditions represents a key mechanism for plant survival during periods of water scarcity, as demonstrated in *Barbacenia* species (Garcia, 1997; Suguiyama et al., 2014; Vieira et al., 2022). Interestingly, the two species of Velloziaceae growing in close proximity in the *campos rupestres* can exhibit distinct traits throughout the dry and rainy seasons. *Barbacenia gentianoides*, for instance, undergoes progressive leaf desiccation during the dry season, followed by regrowth with the onset of rainfall. In contrast, *Vellozia caruncularis* displays traits characteristic of desiccation-tolerant species, maintaining its leaf structure despite extreme dehydration and reactivating metabolic activity during the rainy seasons.

Using an ecophysiological and metabolic framework, this study aimed to elucidate the mechanisms underlying the survival strategies of two co-occurring Velloziaceae throughout the seasonal cycle under natural field conditions. We hypothesized that: (i) leaf metabolic traits underlie the distinct water-use strategies exhibited by each species in different seasons; (ii) leaf senescence in *Barbacenia gentianoides* is offset by investment in leaf resprouting during the rainy season; (iii) in *Vellozia caruncularis*, carbon is preferentially reallocated toward the biosynthesis of protective compounds that preserve leaf viability during dehydration, rather than toward storage compounds for new leaf production, as observed in *B. gentianoides.*

Comparing the responses of *B*. *gentianoides* and *V*. *caruncularis* to water scarcity offers a unique opportunity to investigate differences in water-use strategies, carbon assimilation rates, and metabolic pathways in two species coexisting in close proximity under the same natural environmental conditions. Moreover, understanding how these related species diverge in their drought responses may help elucidate adaptive evolution and species competition within shared habitats. Their distinct resilience strategies could also enhance predictions regarding community responses and the impact of climate change on plant populations.

## Materials and methods

### Area of study and plant material

The study was conducted in an area of *campos rupestres* in Serra do Cipó National Park, Minas Gerais, Brazil (19° 12’ and 19° 34’ S, 43° 27’ and 43° 38’ W), where the highest diversity of the Velloziaceae family occurs. Previous field observations and studies have classified *Barbacenia gentianoides* Goethart & Henrard as desiccation-sensitive (drought tolerant), occurring on exposed rock surfaces (Garcia, 1997) and *Vellozia caruncularis* Mart. ex Seub, as desiccation-tolerant (Alcantara et al., 2015), exhibiting a dead appearance in the dry season (a resurrection species) and occupying microhabitats with deep sand soils. Visual examinations were conducted to characterize the functional traits of both species across the rainy and dry seasons in their natural habitats. The on-site measurements were conducted in 2019, encompassing the rainy season (RS, February), beginning of the dry season (BDS, May), dry season (DS, July), end dry of the season (EDS, early September), and the beginning of the rainy season (BRS, October). Twenty plants of each species with similar size and appearance were selected and standardized for the study. To ensure diversity and minimize spatial interference, we sampled individuals 50m far from each other across the populations, preventing the sampling of clones. Leaves at equivalent positions in plants of both species were chosen for analysis. For *B. gentianoides*, as the desiccated leaves did not recover during the rainy season, analyses were performed on newly resprouted leaves. Leaf fragments from marked plants of each species were carefully collected and promptly stored on dry-ice for subsequent biochemical analyses. During the experimental period, air temperature (°C), relative humidity (RH%), and daily precipitation (mm) data were obtained from the Conceição do Mato Dentro Meteorological Station (Code: 83589, 19° 1’ 13” S and 43° 26’ 2” W).

### Leaf water status and PSII photochemical activity

To assess seasonal physiological responses, water status and photochemical activity were evaluated under field conditions. Leaf relative water content (RWC) was evaluated in fresh leaves from ten plants per treatment. Leaves were collected and weighed to determine fresh weight (FW), then hydrated in distilled water for 24 hours and reweighed to obtain turgid weight (TW). Dry weight (DW) was measured after oven-drying the leaves at 65 °C for 72 hours. The RWC was calculated according to the equation: (FW−DW)/(TW−DW) x 100. Leaf fragments collected in the field were immediately frozen in dry-ice and stored for subsequent analysis of osmotic potential (Ψs). Ten μL of sap were extracted from defrosted leaves in a pressure pump and the Ψs was measured using a model 5520 vapor pressure osmometer (VAPRO). For the determination of leaf electrolyte leakage, 15 fresh leaf discs (0.5cm diameter) from each species were immersed in tubes containing 10 mL of deionized water at room temperature for 12h, then subsequently boiled at 100°C for 25min and cooled to room temperature. The electrical conductivity of the solution was measured in millivolts using a conductivity meter, and the relative values (%) of electrolyte leakage were calculated using the equation: REE= (C1/C2) x 100, where C1 represents the electrical conductivity after incubation at 25°C for 12h and C2 denotes the electrical conductivity after incubation at 100°C for 25min (Zhang et al., 2011). Instantaneous measurements of maximum quantum efficiency of photosystem II (Fv/Fm), chain electron transport rate (ETR) and non-photochemical quenching (NPQ) were performed in plants under clear-sky conditions. Before each measurement, the leaves were dark-adapted for 1h with leaf clips. The measurements were carried out through a 1 μmol m^–2^ s^–1^ light pulse emission to obtain initial fluorescence (F0), followed by a saturating light pulse of 2000 μmol m^–2^ s^–1^ at 650 nm to ensure maximum fluorescence (Fm) using a PAM-210 fluorometer (Walz, Effeltrich, Germany).

### Oxidative damage indicators

The superoxide anion (O_2_•^–^) production was measured in ten plants by monitoring the nitrite formation from hydroxylamine in the presence of O_2_•^–^ (Wu et al., 2010). An aliquot (200 mg) of powdered leaves was mixed with 1.5ml of 65 mM potassium phosphate buffer (pH 7.8) containing 1% (w/v) polyvinylpolypyrrolidone (PVPP). Aliquots of 125 µl of the supernatants were mixed with 125 µl of 65 mM potassium phosphate buffer and 25 µl of 10 mM hydroxylamine hydrochloride and incubated at room temperature for 30min, in darkness. Equal parts of the extract were mixed with Griess reagent, resulting absorbances were read at 540 nm in a microplate reader (Antoniou et al., 2018), and nitrite concentrations were calculated based on a sodium nitrite standard curve. The H_2_O_2_ content was estimated using 200 mg of powdered leaves ground with 0.1% trichloroacetic acid. An aliquot of 300 μL was mixed with 300 μL phosphate-buffered saline (PBS; pH 7.0, 10 mM) and 800 μL of 1 M KI and the H_2_O_2_ content was determined at 390 nm (Moura and Vieira, 2020). The malondialdehyde content (MDA) was determined by homogenizing 80 mg of powered leaves with 1ml of 80% ethanol, with incubation with 20% trichloroacetic acid (TCA) and 0.01% hydroxytoluenebutylate, with or without 0.5% thiobarbituric acid (TBA), at 95 °C for 25min. Samples were centrifuged and the supernatant absorbance was measured at 440 nm, 532 nm (absorbance of specific MDA), and 600 nm (absorbance of non-specific MDA) (Hodges et al., 1999).

### Antioxidant compounds analysis

Ascorbate (Asc) was determined in 100 mg of powdered leaves ground with 1.2ml of 6% TCA previously frozen (Kampfenkel et al., 1995). The dehydroascorbate (DHA) levels were obtained by reducing DHA to Asc through dithiothreitol (DTT). The difference between the total ascorbate and Asc corresponds to DHA content. Total glutathione extraction was done in powdered leaves (100 mg) with 5% (w/v) sulfosalicylic acid (Israr et al., 2006). For oxidized glutathione (GSSG) assays, aliquots of 100 µl of the extracts were added to 900 µl of 0.5 mM sodium EDTA, 50 µl of 0.3 mM 5,5′dithio-bis-(2-nitrobenzoicacid) (DTNB) and 50 µl of 0.5 mM NADPH, all diluted in 100 mM potassium phosphate buffer (pH 7.0). Reactions were started with the addition of 1 µl of glutathione reductase and were kept under light for 20min. Absorbances were read at 412 nm, and estimations were made based on a reduced glutathione (GSH) standard curve. The GSH content was calculated by subtracting the GSSG from total glutathione content. The levels of α-tocopherol and γ-tocopherol were analyzed in 50 mg of powdered leaves subjected to three successive extractions in 3ml of methanol: chloroform (2:1, v/v) containing butylated hydroxytoluene (0.01% w/v) (Yang et al., 2011). The samples were injected in an Agilent 1100 HPLC and compounds separated using an Agilent Eclipse XDB-C18 column (4.6 × 150mm length; 5 µm particle size) and a solvent system consisting of methanol: water (95:5, v/v) with a 1.5ml min^−1^ flow rate. Tocopherols were detected and quantified by fluorescence with excitation at 292 nm and emission at 330 nm. Quantification was based on the fluorescence signal and compared with a calibration curve with authentic standards (Sigma-Aldrich).

### Determination of chlorophyll and carotenoid content

Chlorophylls *a*, *b* were quantified in fragments of fresh leaves ground with 80% acetone using a mortar in the dark and the supernatant obtained was analyzed at 480, 645 and 663 nm (Lichtenthaler and Wellburn, 1983). The extraction and measurement of carotenoids were realized according to Kang et al. (2017). Carotenoids were measured in 100 mg of powdered leaves mixed with 5ml of acetone containing 0.01% butylated hydroxytoluene, sea sand, Na_2_SO_4_, and NaHCO_3_. The resultant extract was centrifuged, and the supernatant was concentrated under vacuum and re-dissolved in CH_2_Cl_2_: acetone (1:1 v/v, 200ml). The solution was filtered through a 0.45mm membrane filter (Whatman, PTFE, 13mm) and analyzed on an Agilent 1100 HPLC system (Hewlett-Packard). The standard or sample solution (20 µl) was injected directly onto a YMC C30 carotenoid column (3 µM, 4.6 x 250 mm) with solvent A (methanol: tert-butylmethyl ether (MTBE): H_2_O, 81:15:4, v/v) and solvent B (MeOH: MTBE: H_2_O, 6:90:4, v/v). A step-gradient elution of 100% solvent A was used for the first 15min, followed by a gradient from 100% solvent A to 100% solvent B over the next 35min. The eluent was detected at 450 nm on a UV-Vis detector. An external calibration method was used for carotenoid quantification. Standards of α-carotene, β-carotene and lutein (Sigma-Aldrich) were used to compare peaks and the respective retention times.

### Carbohydrate analysis

Leaf sections (including tip + middle and base) were lyophilized, powdered and submitted to extraction of soluble sugars (Bispo and Vieira, 2022). An aliquot of 200 mg was extracted three times in boiling 80% ethanol. After centrifugation, the resulting ethanolic supernatants were combined and considered as the soluble-sugar extracts and utilized to determine total soluble sugars using the phenol-sulfuric acid method (DuBois et al., 1956). Starch was quantified from 10 mg of the residue remaining after soluble carbohydrate extraction (Amaral et al., 2007). Starch hydrolysis was carried out in two sequential steps. Initially, 60 units of α-amylase from *Bacillus licheniformis* were used twice in 0.5ml of 10 mM 3-(N-morpholino) propanesulfonic acid buffer (pH 6.0) at 75 °C for 45min. This was followed by two digestion reactions with 15 units of amyloglucosidase from *Aspergillus niger* in 0.5ml of 100 mM sodium acetate buffer (pH 4.5) at 50°C for 30min. The reaction was stopped by adding 100 μl of 800 mM perchloric acid and centrifuged at 10,000 × g for 2min. Starch content was estimated in 50 μl of the supernatant by quantifying the glucose released using 750 μl of Glucose PAP Liquiform reagent (Labtest, Brazil), and incubating at 30°C for 15min. Absorbance was read at 490 nm using a microplate reader. Glucose standards were prepared using glucose from Sigma-Aldrich. Sucrose phosphate synthase (SPS) was extracted as described by Liu et al. (2013). Briefly, 200 mg of frozen leaf tissue was homogenized in cold extraction buffer (50 mM Tris-HCl, pH 7.5; 1 mM EDTA; 1 mM MgCl_2_; 12.5% glycerol; 10% PVP; 10 mM mercaptoethanol). The homogenate was centrifuged at 15,000×g for 20 min at 4°C. SPS activity was measured in a reaction buffer containing 12 mM UDP-glucose, 40 mM fructose-6-phosphate, 200 mM Tris-HCl (pH 7.0), 40 mM MgCl_2_, and 200 μL of extract. The reaction was incubated at 30°C for 30min and stopped with 100 μl of 2 M NaOH, followed by heating at 100°C for 10min. After cooling, samples were mixed with 1ml of 0.1% resorcinol in 95% ethanol and 3.5ml of 30% HCl, then incubated at 80°C for 10min. Sucrose content was determined colorimetrically at 480 nm using a standard curve. ADP-glucose pyrophosphorylase (AGPase) activity was measured in a 300 μl reaction containing 150 μl desalted extract, 80 mM HEPES-KOH (pH 7.9), 2 mM MgCl_2_, 10 mM glucose-1,6-BP, 10 mM phosphoglyceric acid, 5 mM DTT, 0.2 mM NAD^+^, 1 mM ADP-glucose, 10 mM NaF, 1 U/ml phosphoglucomutase, and 2.5 U/ml glucose-6-P dehydrogenase (Müller-Röber et al., 1992). The reaction was initiated with 2 mM sodium pyrophosphate, and NAD^+^ reduction was monitored at 340 nm. Protein concentration was determined using the Bradford method (1976). β-amylase activity was assayed as described by Li-xu et al. (2013), with α-amylase inhibited using 0.1 M EDTA (pH 3.4). The reaction used 0.1 mM citrate buffer (pH 3.4), 2% soluble starch, and 0.7ml of enzyme extract in a 2ml volume. Samples were incubated at 30°C for 5min, reactions stopped with 2ml DNS, and absorbance measured at 540 nm.

### Metabolic profiling

The metabolic profiling was evaluated using gas chromatography coupled with mass spectrometry (GC/MS). Leaf metabolites were extracted from freeze-dried tissue (100 mg) using a methanol, chloroform, and water solvent mixture (2:1:2 v/v/v). Adonitol served as the internal standard. The resulting concentrate from vacuum drying was subjected to derivatization following the method outlined by Lisec et al. (2006). The derivatized metabolite samples were then injected into an Agilent 7890 gas chromatograph (Agilent, CA, USA) coupled to a 5977mass spectrometer. Peak detection and deconvolution were conducted using MassHunter ChemStation software v. B.06.00 (Agilent) and matched against the National Institute of Standards and Technology (NIST) Library, and authentic standards. Subsequently, the data were processed and analyzed using MetaboAnalyst 6.0.

### Endogenous levels of stress-related phytohormones

The extraction and analysis of endogenous contents of abscisic acid (ABA), jasmonic acid (JA), and salicylic acid (SA) were conducted following the methods outlined in Dobrev and Vankova (2012) and Djilianov et al. (2013). A 100 mg aliquot of powdered leaves was subjected to a cold extraction buffer (500 µl) containing methanol:water:formic acid (15:4:1, v/v/v). Subsequently, a mixture of stable isotope-labelled internal standards (10 *p*mol/sample): [^2^H_6_] ABA (Olchemin, Ltd); [^2^H_5_] JA (C-D-N Isotopes Inc.); and [^2^H_4_] SA (Sigma-Aldrich) were added to the plant homogenates. Reversed-phase and ion-exchange chromatography (Oasis-MCX, Waters) resulted in a fraction eluted with methanol. Fractions were evaporated to dryness in a vacuum concentrator and dissolved in 30 µl of 10% methanol. An aliquot (10 μL) was analyzed by HPLC with Ultimate 3000 (Dionex, Sunnyvale) coupled to a hybrid triple quadrupole-linear ion trap mass spectrometer (3200 Q TRAP, Applied Biosystems) set to selected reaction-monitoring mode. The mass spectrometer was set at electrospray ionization mode, and the following ion source parameters were used: ion source voltage -4,000 V (negative mode); nebulizer gas 50psi; heater gas 60psi; curtain gas 20psi; heater gas temperature 500 °C. The phytohormones were quantified using the isotope dilution method with multilevel calibration curves. Measurement of endogenous precursor of ethylene, 1-aminocyclopropane-1-carboxylic acid (ACC), was performed as described by Gao et al. (2013). Fresh leaf samples were harvested, ground to a fine powder in liquid nitrogen and mixed with 750 µl of cold extraction buffer (80:19:1 methanol: water: acetic acid, v/v/v). After shaking for 16h at 4 °C in the dark, the supernatants were collected. Filtrates were dried using nitrogen gas at room temperature and were then dissolved in 200 µl of methanol. For quantification, an aliquot of dissolved sample was analyzed using an Applied Biosystems MDS SCIEX 4000 QTRAP liquid chromatography-tandem mass spectrometry system (AB Sciex, Foster, CA, USA). ACC standards (Sigma-Aldrich) were used for the quantitative analyses.

### Data analysis

Climatic data variation (rainy season x dry season) was considered as a factor (independent variable), and the physiological and biochemical responses as variables (dependent variable). All data were subjected to One-way ANOVA (p < 0.05) and compared by Tukey´s test using R environment with the assistance of the RStudio 1.3.1 interface. The metabolic data matrix was subjected to hierarchical clustering heatmap analysis, principal component analysis (PCA) and enrichment analysis. The metabolic data were analyzed using the free web-based metabolomics tool MetaboAnalyst 6.0 (https://www.metaboanalyst.ca).

## Results

### Leaf water status and photochemical activity

**Figure 1:**
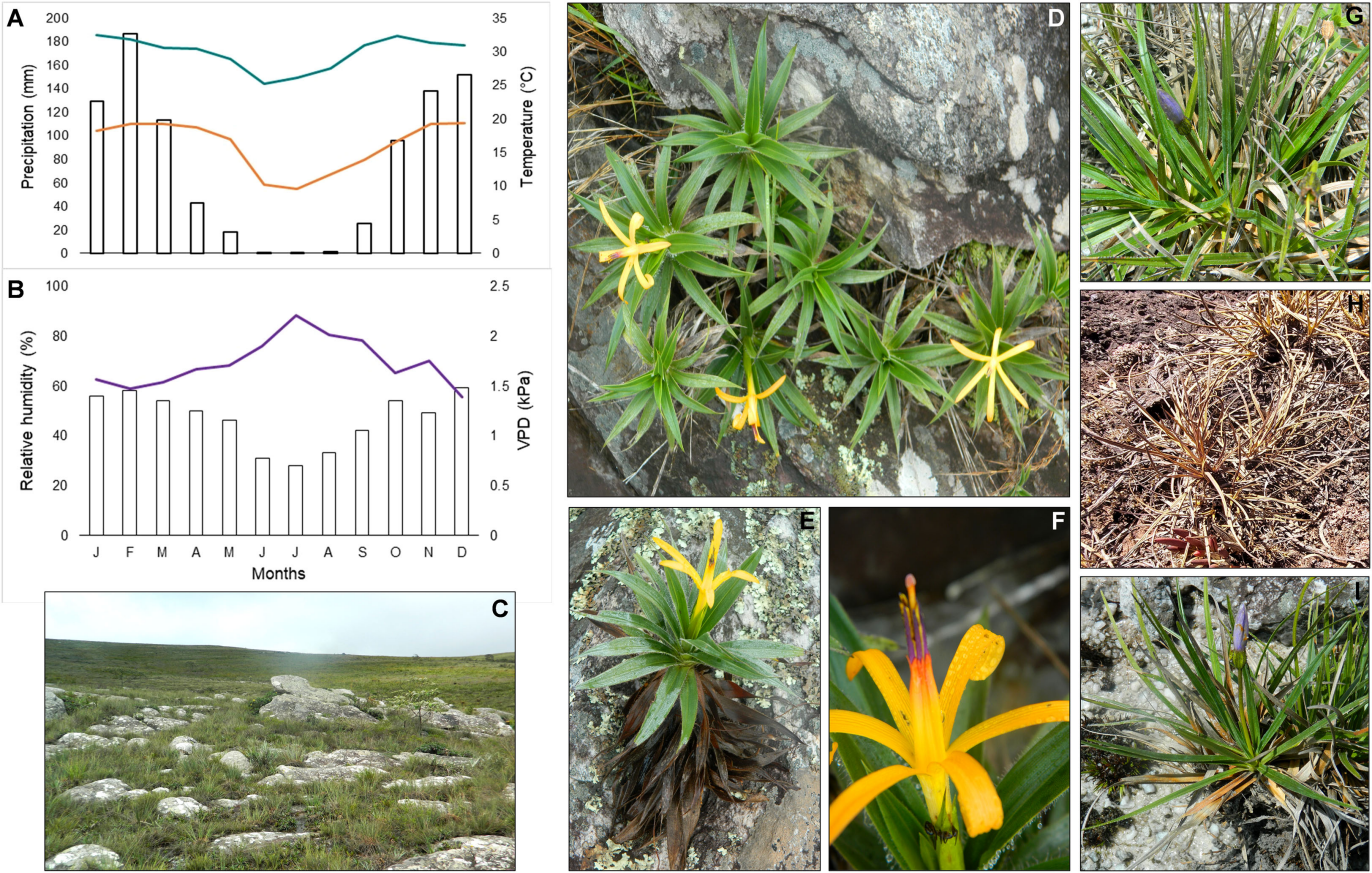
Climatic data and plant species naturally growing in the *Campos rupestres*, Serra do Cipó, MG, Brazil. **(A)** Mean annual precipitation (mm) (bars) and maximum and minimum temperatures (°C) (lines); **(B)** Relative humidity (%) (bars) and vapor pressure deficit (VPD) (line); **(C)** Rock outcrops characteristic of *Campos rupestres*; **(D–F)** *Barbacenia gentianoides* and its distribution in rock fissures, displaying both new and senescent leaves and flower details; **(G)** *Vellozia caruncularis* in a hydrated state during the rainy season, **(H)** in a fully desiccated state during the dry season, and **(I)** in a recovered state at the beginning of the rainy season.

Seasonality in the study area was well-defined, characterized by a marked decline in precipitation, temperature, and relative humidity during the dry season (DS, July), as shown in **Figures 1A-C**. These changes increased vapor pressure deficit (VPD). *Barbacenia gentianoides* exhibited a progressive dehydration gradient from the leaf apex to the base, reaching its peak at the end of the dry season (EDS, September). Although most leaves senesced, their basal portions remained viable. However, most of these desiccated leaves failed to recover during the subsequent rainy season (RS, February) (**Figures 1D–F**), with only a few exhibiting partial revival. Nevertheless, most *B. gentianoides* individuals could resprout and produce new leaves after the beginning of the rainy season (BRS, October). *Vellozia caruncularis* displayed a fully desiccated appearance by the EDS, characterized by a complete loss of leaf pigmentation. In contrast, during the RS, younger leaves retained higher hydration levels (**Figures 1G, H**). All individuals observed in the field showed partial recovery of water status at the BRS, followed by complete rehydration by December of the same year (**Figure 1I**).

**Figure 2:**
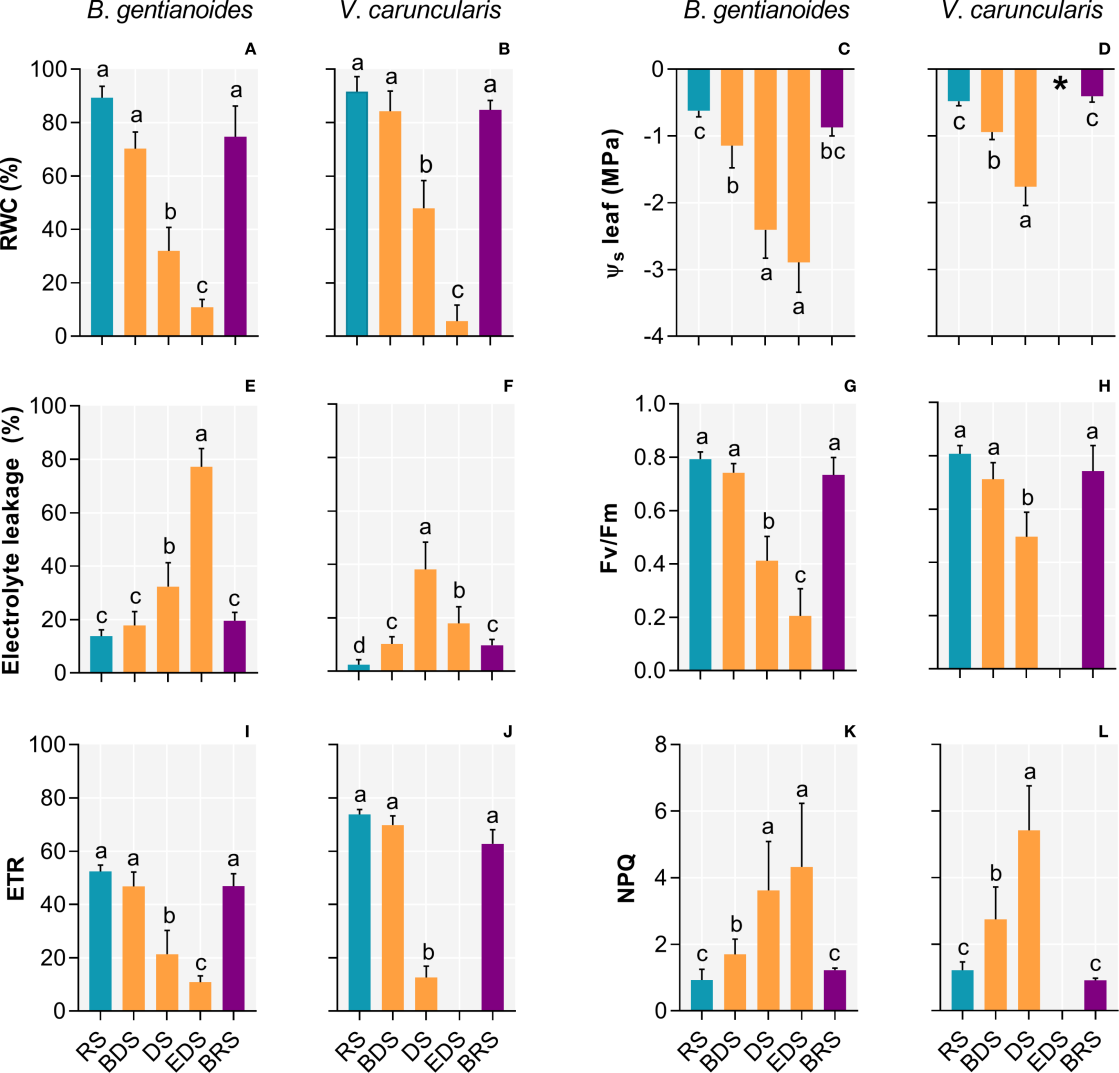
Ecophysiological parameters in the co-occurring species *B*. *gentianoides* and *V*. *caruncularis* growing under field conditions during the seasonal cycle. **(A, B)** Relative leaf water content (%); **(C, D)** Leaf osmotic potential (MPa); **(E, F)** Electrolyte leakage (%); **(G, H)** Quantum efficiency of photosystem II (Fv/Fm); **(I, J)** Electron transport rate and **(K, L)** non-photochemical quenching (NPQ). RS, rainy season; BDS, beginning of the dry season; DS, dry season; EDS, end of the dry season; BRS, beginning of the rainy season (n = 10). Lowercase letters indicate a significant difference (one-way ANOVA, Tukey test *P*<0.05) among the distinct seasons.

Differences in the leaf water status between the two species confirm their contrasting water-use strategies (**Figure 2**). During the DS, *B*. *gentianoides* gradually declined in RWC, reaching 32%. In contrast, *V*. *caruncularis* initially maintained higher leaf water levels during the same period (48%) but subsequently experienced a severe decline to 5.6% by the EDS (**Figures 2A, B**). Between BDS and EDS, both species primarily maintained water status through osmotic regulation. Despite accumulating lower levels of solutes than *B. gentianoides* (**Figures 2C, D**), *V*. *caruncularis* demonstrated greater efficiency in maintaining RWC during the DS (**Figures 2A, B**). By the EDS, osmotic potential could not be measured in *V*. *caruncularis* due to complete leaf desiccation (**Figures 1H**, **2D**). At BRS, both the resprouted leaves of *B. gentianoides* and the previously desiccated leaves of *V. caruncularis* recovered to RWC and osmotic potential values comparable to those observed at RS (**Figures 2A–D**). Despite exhibiting effective osmotic adjustment, *B. gentianoides* showed significantly higher electrolyte leakage during the DS, whereas *V. caruncularis* maintained more effective water-status regulation throughout progressive leaf desiccation, delaying metabolic shutdown until the EDS (**Figures 2E, F**).

Changes in water status markedly influenced photochemical performance in both species. The Fv/Fm remained near optimal levels until the BDS, but declined sharply thereafter, indicating increasing photoinhibition during the DS (**Figures 2G, H**). Concurrently, the electron transport rate (ETR) decreased significantly throughout the DS in both species (**Figures 2I, J**), while non-photochemical quenching (NPQ) progressively increased in response to leaf dehydration (**Figures 2K, L**). By the EDS, photochemical activity in *V*. c*aruncularis* was suppressed entirely. However, all parameters gradually recovered upon rehydration at BRS. Similarly, resprouted leaves of *B*. *gentianoides* exhibited fully restored photochemical function during this period (**Figures 2G–L**).

### Oxidative metabolism and pigments

**Figure 3:**
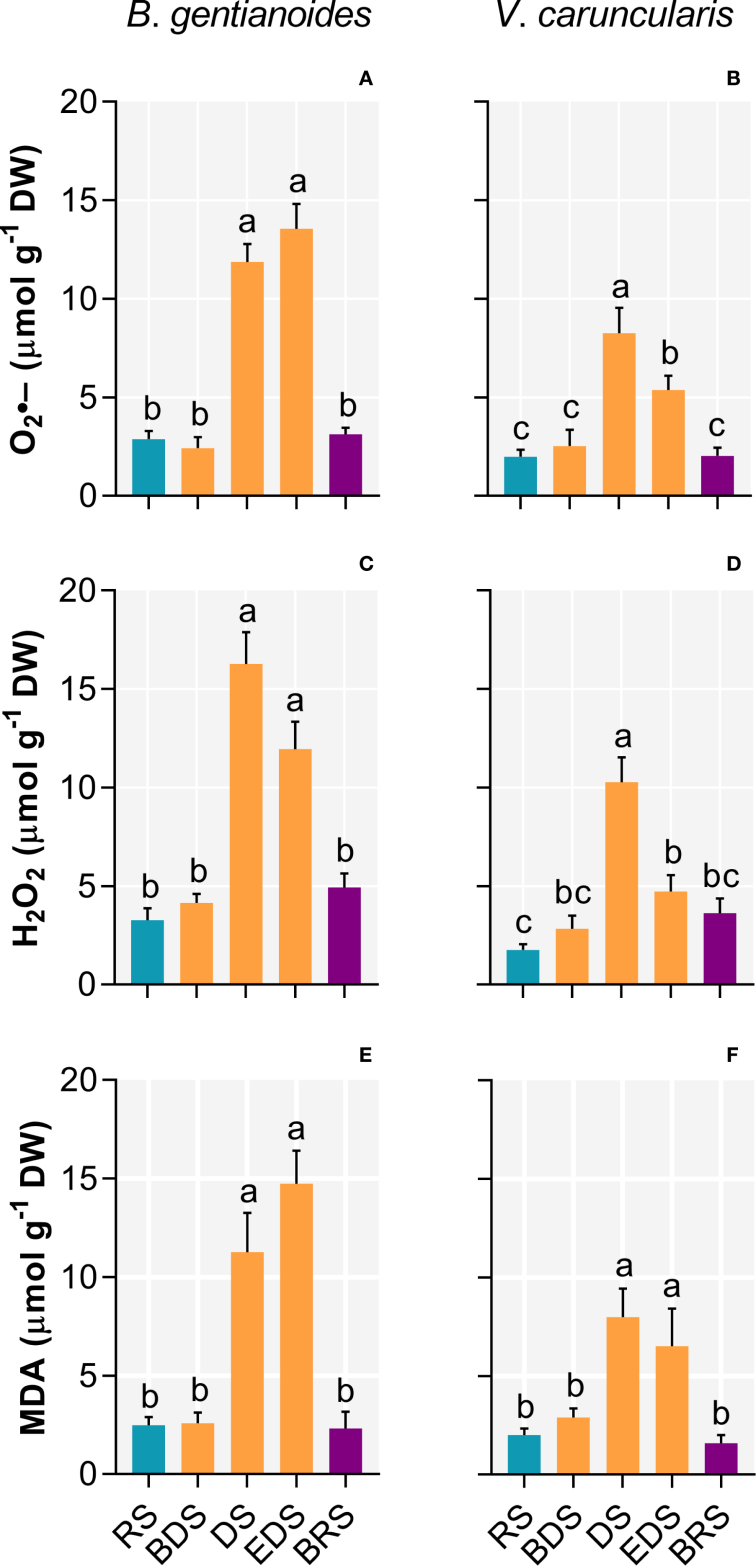
Oxidative damage indicators in the co-occurring species *B*. *gentianoides* and *V*. *caruncularis* growing under field conditions during the seasonal cycle. **(A, B)** Anion superoxide (O2˙^–^); **(C, D)** hydrogen peroxide (H_2_O_2_) and **(E, F)** malondialdehyde (MDA) concentrations. RS, rainy season; BDS, beginning of the dry season; DS, dry season; EDS, end of the dry season; BRS, beginning of the rainy season (n = 10). Lowercase letters indicate a significant difference (one-way ANOVA, Tukey test P<0.05) among the distinct seasons.

Progressive leaf dehydration and the onset of photoinhibition during the DS led to pronounced increases in oxidative damage-inducing indicators in both species (**Figure 3**). In *B. gentianoides*, superoxide (O_2_•^–^) production increased 313.5% during the DS and 372% by the EDS (**Figure 3A**), while *V. caruncularis* showed increases of 314.5% and 169.8%, respectively (**Figure 3B**). Likewise, hydrogen peroxide (H_2_O_2_) accumulation was consistently higher in *B. gentianoides* throughout dehydration compared to *V. caruncularis* (**Figures 3C, D**). Disruption of the photochemical apparatus and increased oxidative stress were further evidenced by substantial increases in foliar malondialdehyde (MDA) levels: 494% in *B*. *gentianoides* and 225.5% in *V*. *caruncularis* from DS to EDS (**Figures 3E, F**).

**Figure 4:**
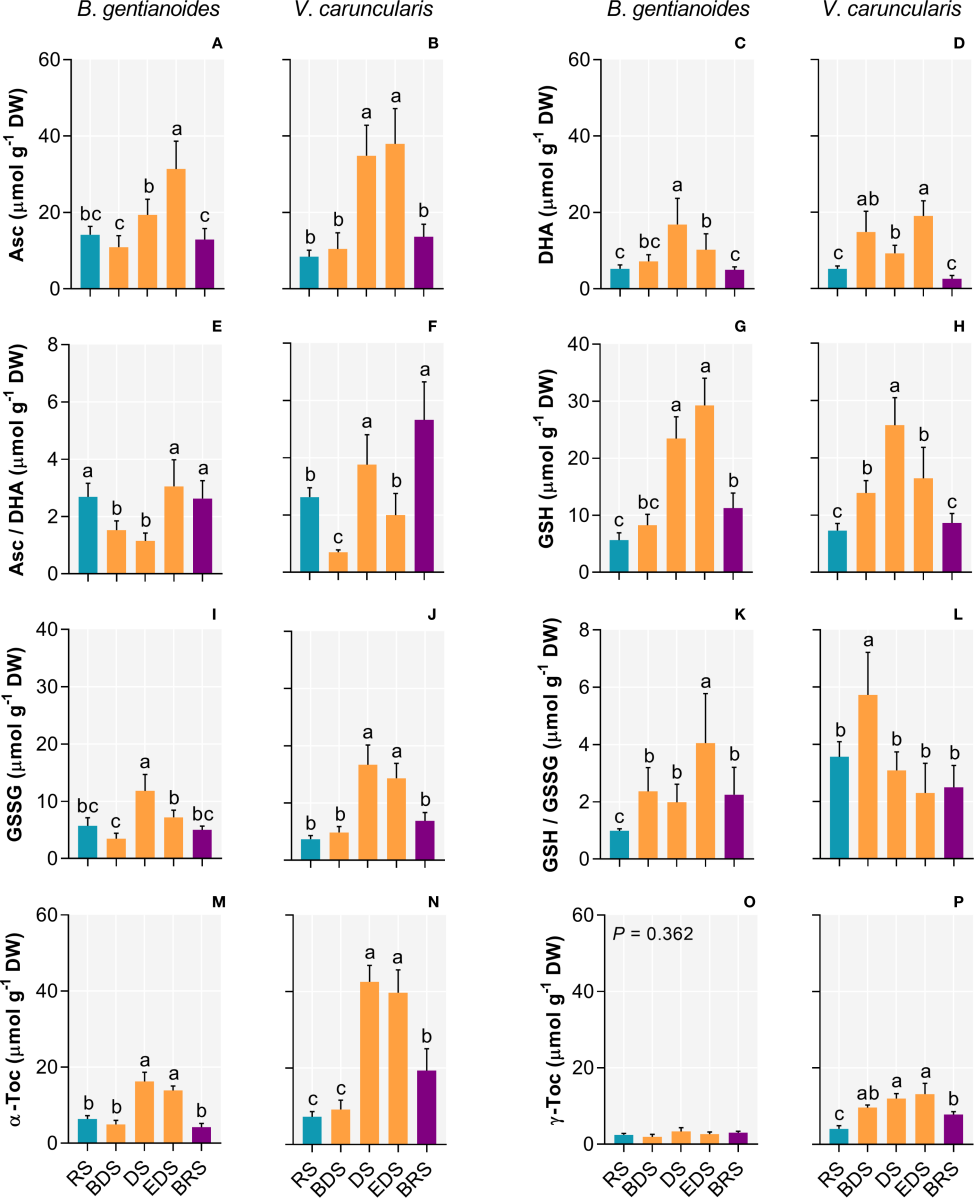
Antioxidant compounds in the co-occurring species *B*. *gentianoides* and *V*. *caruncularis* growing under field conditions during the seasonal cycle. **(A, B)** Ascorbate (Asc); **(C, D)** Dehydroascorbate (DHA); **(E, F)** Asc/DHA ratio **(G, H)** Reduced glutathione (GSH); **(I, J)** Oxidized glutathione (GSSG); **(K, L)** GSH/GSSG ratio; **(M, N)** α-Tocopherol and **(O, P)** γ-Tocopherol concentrations. RS, rainy season; BDS, beginning of the dry season; DS, dry season; EDS, end of the dry season; BRS, beginning of the rainy season. (n = 10). Lowercase letters indicate a significant difference (one-way ANOVA, Tukey test P<0.05) among the distinct seasons.

Between the DS and the EDS, both species maximized the synthesis of antioxidant compounds. At the DS, *B. gentianoides* and *V. caruncularis* showed significant increases in ascorbate (121.2% and 349%, respectively), dehydroascorbate (94.8% and 264.6%), reduced glutathione (419.7% and 252.7%), oxidized glutathione (107% and 353.9%), and α-tocopherol (155% and 491.5%). Additionally, *V. caruncularis* accumulated γ-tocopherol, showing a 222% increase during the DS, whereas this compound was undetectable in *B. gentianoides* (**Figures 4A–P**).

**Figure 5:**
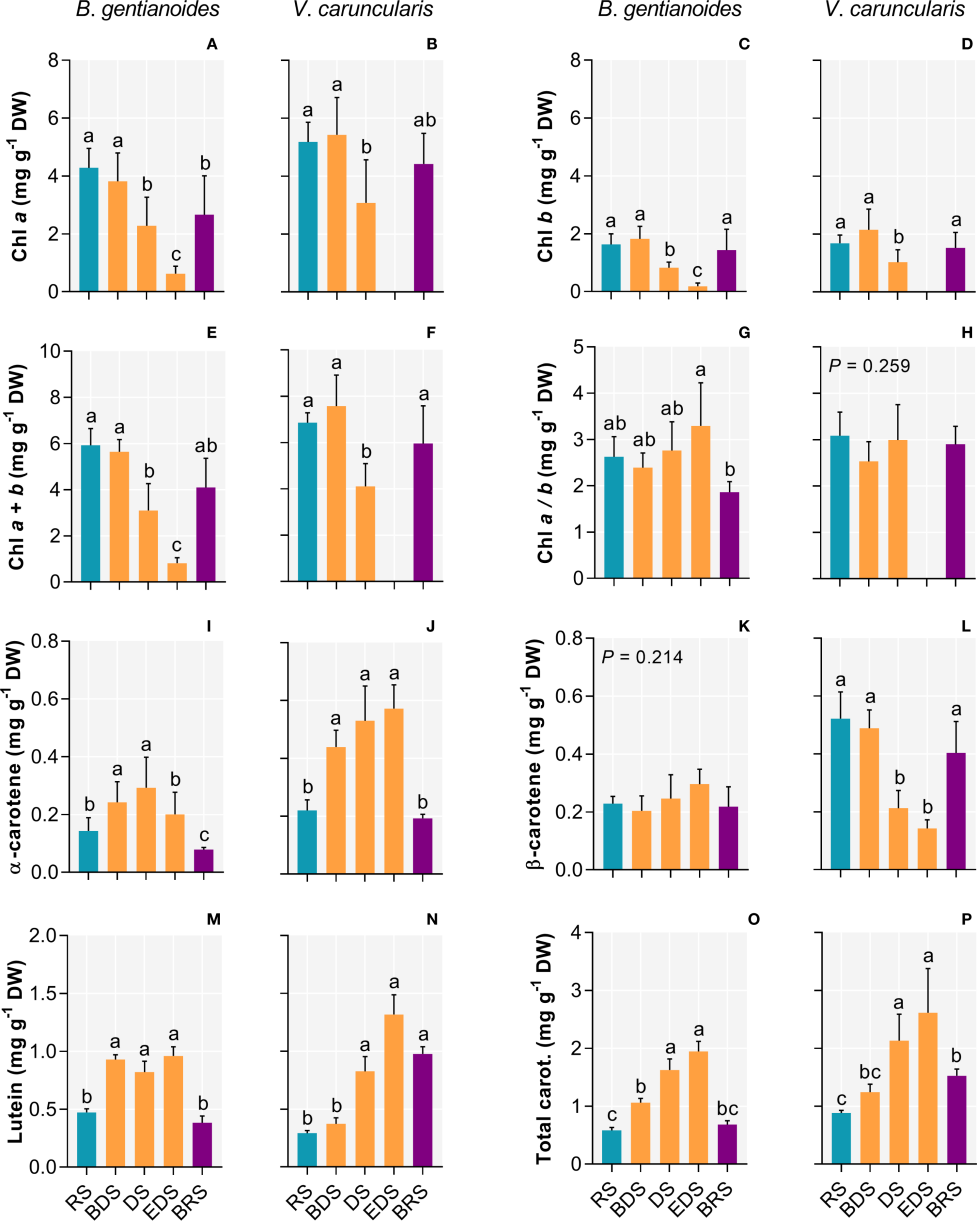
Chlorophylls and carotenoids in the co-occurring species *B*. *gentianoides* and *V*. *caruncularis* growing under field conditions during the seasonal cycle. **(A, B)** Chlorophyll *a* (Chl *a*); **(C, D)** Chlorophyll *b* (Chl *b*); **(E, F)** Chl *a* + Chl *b*; **(G, H)** Chl *a/*Chl *b* ratio; **(I, J)** α-Carotene; **(K, L)** β-Carotene; **(M, N)** Lutein and **(O, P)** Total carotenoids concentrations. RS, rainy season; BDS, beginning of the dry season; DS, dry season; EDS, end of the dry season; BRS, beginning of the rainy season (n = 10). Lowercase letters indicate a significant difference (one-way ANOVA, Tukey test P<0.05) among the distinct seasons.

Both species exhibited a progressive decrease in chlorophyll *a*, *b*, and total chlorophyll content from the DS onward (**Figures 5A–F**), although the chlorophyll a/b ratio changed significantly only in *B. gentianoides* (**Figures 5G, H**). In *B. gentianoides*, chlorophyll levels remained low by the EDS, whereas in *V. caruncularis*, these pigments were completely degraded (**Figures 5B, D, F**). Conversely, from the BDS to the EDS, both species showed substantial accumulation of α-carotene (**Figures 5I, J**), lutein (**Figures 5M, N**), and total carotenoids (**Figures 5O, P**). Notably, changes in β-carotene concentration (**Figures 5K, L**) were detected only in *V. caruncularis*, which exhibited a 73% decrease compared to the RS.

### Carbohydrate metabolism

**Figure 6:**
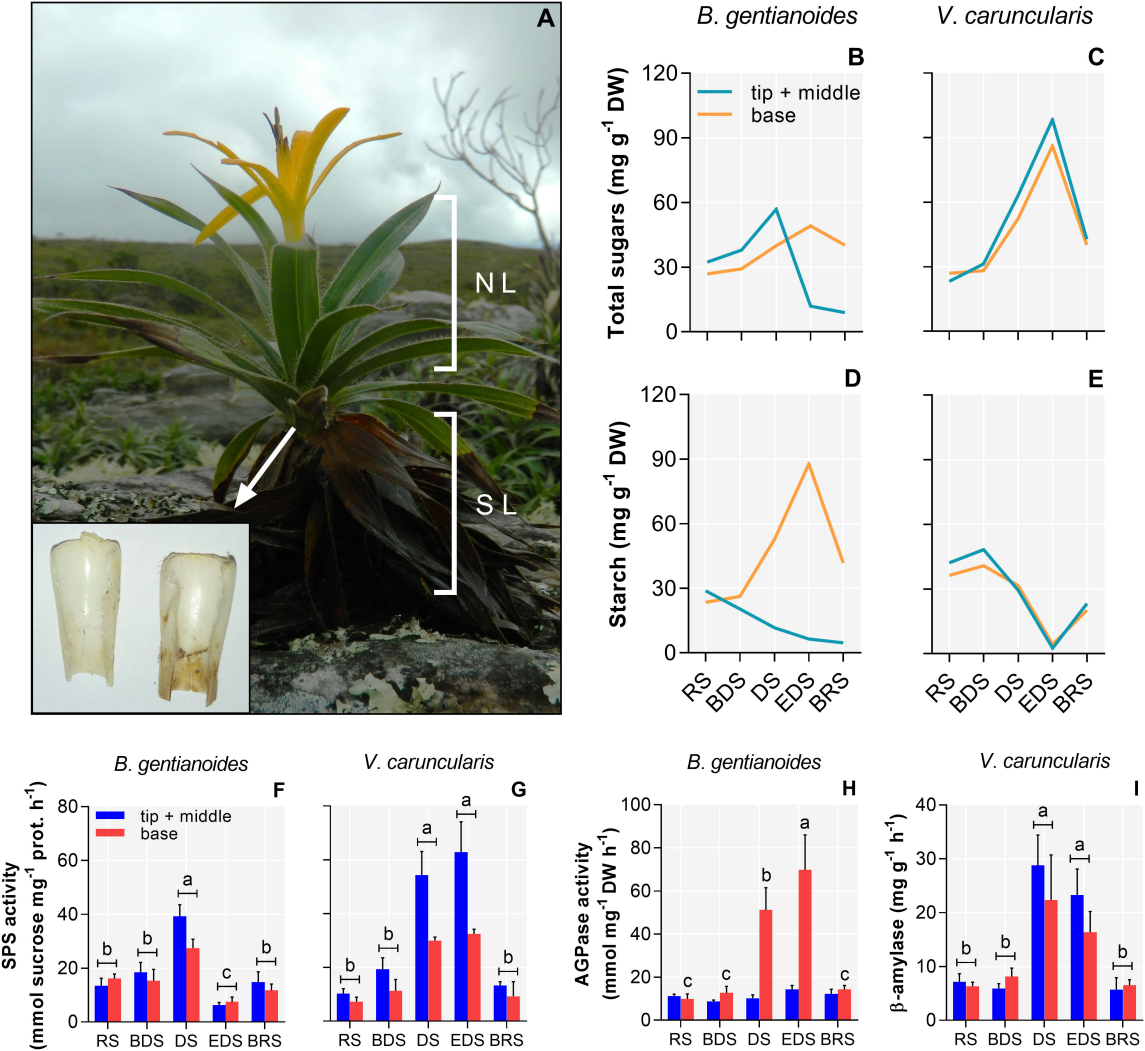
Soluble carbohydrates, starch, and activity of related enzymes in the co-occurring species *B*. *gentianoides* and *V*. *caruncularis* growing under field conditions during the seasonal cycle. **(A)** *B*. *gentianoides* with both young and senescent leaves, and detail of the leaf base where reserves are stored; **(B, C)** Total soluble carbohydrates at the tip + middle and at the leaf base; **(D, E)** Starch at the tip + middle and at the leaf base; **(F, G)** Sucrose phosphate synthase (SPS) activity; **(H)** ADP-glucose pyrophosphorylase (AGPase) activity and **(I)** β-amylase activity. RS, rainy season; BDS, beginning of the dry season; DS, dry season; EDS, end of the dry season; BRS, beginning of the rainy season; NL, new leaves; SL, senescent leaves (n = 10). Lowercase letters indicate a significant difference (one-way ANOVA, Tukey test P<0.05) among the distinct seasons.

In *B*. *gentianoides*, soluble carbohydrate levels declined in the leaf tip and mid-sections during the DS, followed by a marked increase in the leaf base by the EDS (**Figures 6A, B**). Activities of sucrose phosphate synthase (SPS) and ADP-glucose pyrophosphorylase (AGPase) exhibited inverse patterns, particularly at EDS, suggesting sugar remobilization and subsequent starch synthesis in the leaf bases (**Figures 6D, F, H**). Conversely, *V*. *caruncularis* showed increased soluble sugar levels across all leaf regions, likely resulting from starch degradation, as evidenced by increased SPS and β-amylase activities (**Figures 6C, E, G, I**).

### Metabolic profiling

**Figure 7:**
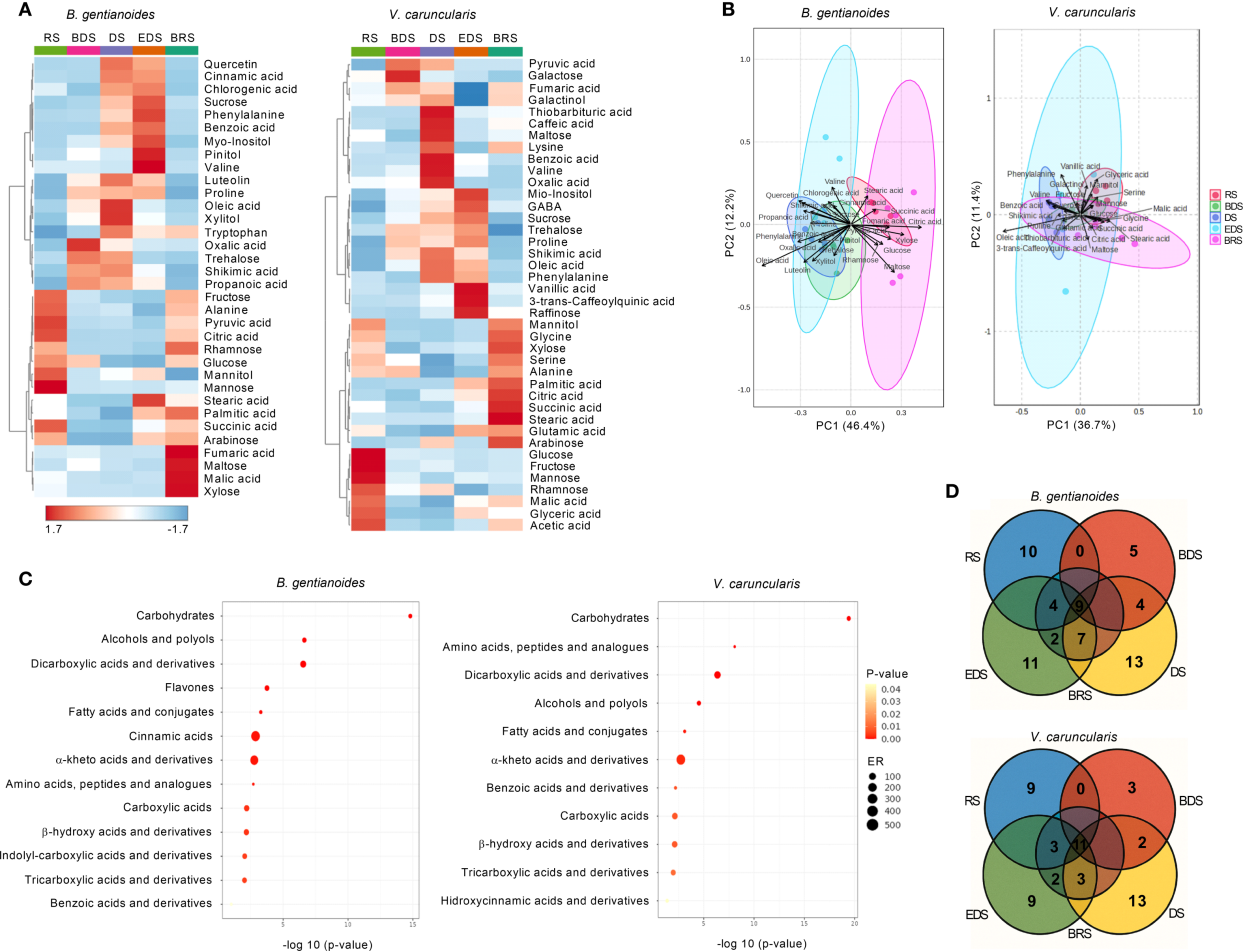
Metabolic profiling in the co-occurring species *B*. *gentianoides* and *V*. *caruncularis* growing under field conditions during the seasonal cycle. **(A)** Comparative heatmap showing metabolic reprogramming during drought acclimation and dehydration stress in both species. The intensity of each metabolite peak for all samples represents the mean of each treatment. Colours represent abundance (red, significantly higher than control; blue, significantly lower than control; lighter, no significant changes) by Tukey’s test (p < 0.05). **(B)** Principal components analysis (PCA) of the metabolic profiling showing the clustering of major compounds responsive to the dehydration–rehydration cycle. **(C)** Enrichment analysis identifying key compound groups associated with drought acclimation; **(D)** Venn diagrams displaying the overlap of upregulated compounds at each time point across different seasons. RS, rainy season; BDS, beginning of the dry season; DS, dry season; EDS, end of the dry season; BRS, beginning of the rainy season; ER, enrichment ratio (n = 10).

Between the BDS and the EDS, dehydration-tolerance-related sugars, including sucrose, trehalose, myo-inositol, and pinitol, increased in *B. gentianoides* (**Figure 7A**). In contrast, monosaccharides such as glucose, fructose, mannose, and arabinose peaked during the RS and progressively decreased with advancing dehydration. Notably, maltose and xylose accumulated to high levels in resprouted leaves of *B. gentianoides* at the BRS. In *V*. *caruncularis*, galactose increased during the BDS, while galactinol and trehalose increased steadily from the BDS and DS to the EDS. Maltose levels also peaked during the DS, whereas myo-inositol, sucrose, and raffinose were more prevalent at the EDS, with raffinose showing a unique increase during this period. Throughout dehydration, glucose and fructose decreased, while xylose increased predominantly during the rainy periods (BRS and RS). Arabinose peaked during both the DS and BRS. Rhamnose was most abundant during the RS, but declined at BDS, followed by a slight increase during the DS (**Figure 7A**).

Most amino acids peaked between DS and EDS in *B*. *gentianoides*, except for tryptophan which also accumulated at the BRS, and alanine, which peaked at BRS and RS (**Figure 7A**). In *V. caruncularis*, proline, phenylalanine, and GABA, significantly increased during the DS-EDS. In contrast, alanine, serine, and glycine peaked at BRS and RS. Glutamic acid levels increased during EDS and BRS, while lysine increased during DS and BRS, and valine accumulation was exclusive to the DS (**Figure 7A**). In *V*. *caruncularis*, several organic acids associated with central metabolism and stress response accumulated at specific stages of dehydration and rehydration (**Figure 7A**). Shikimic acid levels increased from the BDS to the EDS, while pyruvic and fumaric acids peaked between the BDS and the DS, with the latter also increasing during BRS. Benzoic, caffeic, oxalic, and thiobarbituric acids were detected exclusively during the DS, and vanillic and 3-trans-caffeoylquinic acids were detected only at the EDS (**Figure 7A**). In contrast, acetic and malic acids increased during the RS and BRS, while citric, stearic, and succinic acids peaked at the BRS. Palmitic acid concentrations were elevated between the EDS and the BRS.

In *B*. *gentianoides*, oxalic, propanoic, and shikimic acids were the most abundant from the BDS to the DS, whereas benzoic, cinnamic, and chlorogenic acids primarily accumulated between the DS and EDS. The long-chain fatty acids stearic and palmitic peaked at EDS and BRS, coinciding with key stages of structural reorganization. Notably, the flavonoids luteolin and quercetin increased markedly during the DS and EDS, consistent with their role in antioxidant defense. Organic acids typically involved in respiratory metabolism, such as pyruvic, citric, and succinic acids, reached high levels during the BRS and RS, indicating metabolic reactivation. Additionally, fumaric and malic acids showed significant increases during BRS, further supporting enhanced TCA cycle activity during post-stress recovery (**Figure 7A**).

The metabolic patterns were further explored using principal component analysis (PCA). In *B. gentianoides*, the PCA score scatter plot revealed that PC1 and PC2 accounted for 46.4% and 12.2% of the total variance, respectively. The separation between BDS and EDS was driven primarily by oleic acid, phenylalanine, luteolin, quercetin, trehalose, and xylitol. In contrast, citric acid, maltose, glucose, pyruvic acid, and cell wall-associated sugars were more abundant during the BRS and RS (**Figure 7B**). In *V*. *caruncularis*, PC1 explained 48.1% of the variance, with oleic acid, shikimic acid, benzoic acid, phenylalanine, proline, and sucrose characterizing the dry‐season samples (DS–EDS), and citric acid, succinic acid, stearic acid, glyceric acid, glycine, and mannitol characterizing rainy‐season samples (BRS–RS).

Enrichment analysis revealed distinct metabolic strategies between the two species. In *B. gentianoides*, drought resistance was primarily driven by the accumulation of carbohydrates (particularly alcohols and polyols), flavones, cinnamic acids, and alpha-keto acids. In contrast, desiccation tolerance in *V. caruncularis* was mainly underpinned by the accumulation of carbohydrates, amino acids, peptides, dicarboxylic acids, and fatty acids (**Figure 7C**). The Venn diagram illustrated the number of compounds upregulated at each season and highlights metabolites that consistently remained at high concentrations across successive dehydration stages and throughout the RS in both species (**Figure 7D**).

### Phytohormone changes

**Figure 8:**
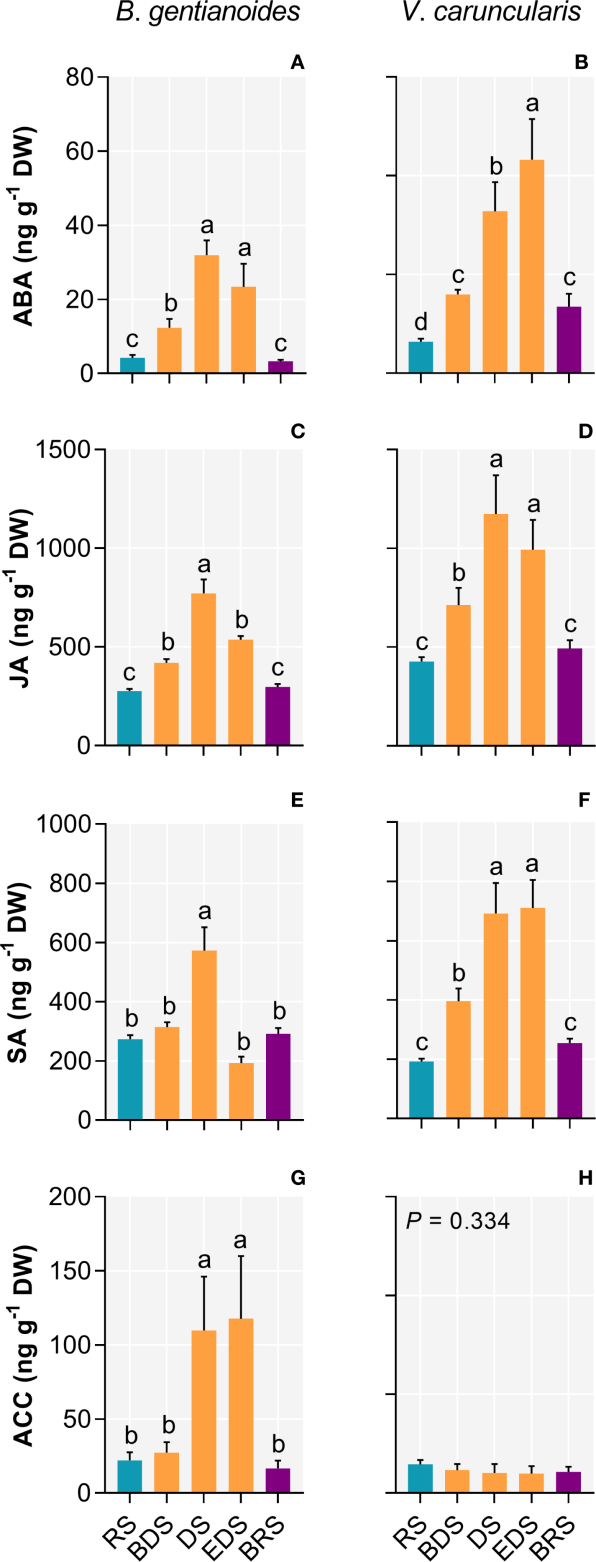
Stress-related phytohormones in the co-occurring species *B*. *gentianoides* and *V*. *caruncularis* growing under field conditions during the seasonal cycle. **(A, B)** Abscisic acid (ABA); **(C, D)** Jasmonate (JA); **(E, F)** Salicylic acid (SA); **(G, H)** Ethylene precursor, 1-aminocyclopropane-1-carboxylic acid (ACC). RS, rainy season; BDS, beginning of the dry season; DS, dry season; EDS, end of the dry season; BRS, beginning of the rainy season (n = 10). Lowercase letters indicate a significant difference (one-way ANOVA, Tukey test *P*<0.05) among the distinct seasons.

In *B*. *gentianoides*, the DS triggered substantial hormone responses, marked by significant increases in ABA (656.3%), JA (179.5%), and SA (109.4%). The ethylene precursor 1-aminocyclopropane-1-carboxylic acid (ACC) peaked at the EDS (429.7%), coinciding with the onset of leaf senescence (**Figures 8A, C, E, G**). Similarly, in *V*. *caruncularis*, ABA increased significantly at the EDS (578.2%), whereas JA accumulation was most significant during the DS (174%). In contrast, SA levels in *V*. c*aruncularis* did not exhibit substantial variation between DS and ED (**Figures 8B, D, F, H**).

## Discussion

### Contrasts in water conservation and usage define different photoprotective strategies

The co-occurring species exhibited contrasting strategies in response to seasonal water scarcity. *Vellozia caruncularis* adopted a desiccation-tolerance strategy, characterized by strict water-loss control and the activation of protective mechanisms that preserve cellular integrity during complete dehydration, enabling rapid functional recovery upon rehydration. In contrast, *Barbacenia gentianoides* displayed desiccation sensitivity, relying on stomatal regulation and osmotic adjustment to sustain moderate tissue hydration and mitigate photodamage during dry periods.

The distinct strategies of water‐loss regulation underscore the critical role of leaf traits in adapting to severe dehydration by modulating carbon assimilation and sustaining productivity. Although significant reductions in relative water content (RWC) were only observed from DS onward, hormonal signaling was already activated at the BDS, as indicated by increased levels of ABA and JA in both species. Yobi et al. (2017) demonstrated that metabolic priming in the resurrection species *Sporobolus stapfianus* begins as RWC declines toward 60%. Still, the hallmark metabolic features of true desiccation tolerance (DT) emerge once this threshold is crossed. In our study, we found that at approximately 60% RWC—the point at which early dehydration responses are triggered—*B. gentianoides* and *V. caruncularis* exhibited strikingly similar physiological and metabolic adjustments, suggesting the existence of a conserved priming phase preceding the divergence into species-specific DT mechanisms. As drought becomes more severe and RWC drops below 20% at the EDS, the responses of the two species begin to diverge markedly. A similar threshold‐dependent response was reported in the resurrection plant *Xerophyta schlechteri*, where RWC values below 35% trigger age‐related senescence in older leaf tissues, while in younger tissues, senescence is inhibited (Radermacher et al., 2019).

From EDS to DS, the progressive decline in photochemical activity in *B. gentianoides* parallels its leaf desiccation and the onset of basal senescence. Although CO_2_ limitation under severe dehydration likely contributes to pronounced photoinhibition—evidenced by reduced electron transport rates (ETR) and elevated non-photochemical quenching (NPQ)—the increase in NPQ suggests that B. gentianoides retains the capacity to dissipate excess light energy as heat. This photoprotective mechanism aligns with the framework proposed by Rios et al. (2023), in which species that sustain high light-use efficiency preserve chlorophyll content throughout the seasons, modulate effective PSII quantum yield (ϕPSII) during the rainy season, and enhance ϕNPQ under drought. By converting excess excitation energy into heat, these plants effectively mitigate photooxidative damage when CO_2_ assimilation is constrained.

In *B. gentianoides*, partial chlorophyll retention and enhanced NPQ sustain minimal photochemical activity, underpinning the metabolic adjustments required for survival and subsequent resprouting at BRS. Conversely, *V. caruncularis* maintains optimal Fv/Fm, ETR, and NPQ until the DS, after which it actively degrades chlorophyll, suspends assimilatory metabolism, and enters an anabiotic state under severe water deficit and high irradiance at the EDS. This desiccation-tolerance strategy prevents irreversible oxidative damage to the photosynthetic apparatus during recurrent desiccation events (Aidar et al., 2014; Rakić et al., 2015; Vieira et al., 2022).

Through dehydration, antioxidant defense metabolism remained active in both species, contributing to minimizing PSII damage. Increased levels of ascorbate, dehydroascorbate, and glutathione (oxidized and reduced forms) supported ROS scavenging. Vieira et al. (2024) demonstrated that these antioxidants help maintain oxidative damage within tolerable levels in *Barbacenia graminifolia*. Moreover, increased tocopherol concentration, particularly in *V. caruncularis* from DS to BRS, further enhanced photoprotection by scavenging free radicals and O_2_•^–^, acting synergistically with other antioxidant systems. Tocopherol may also contribute to photoprotection by modulating thylakoid membrane permeability to protons, thus promoting lumen acidification and activating violaxanthin de-epoxidase (Munné-Bosch and Alegre, 2002; Vieira et al., 2024). These responses underscore the tight synchronization between dehydration and photoprotective mechanisms in both species.

Elevated total carotenoids (primarily α-carotene and lutein), combined with reduced or absent chlorophyll, especially during DS and EDS, protect the photosynthetic apparatus from excess light and indicate oxidative stress in both species. Carotenoids dissipate excess excitation energy as heat through NPQ or via chemical quenching mechanisms, yielding oxidized derivatives that function as stress sensors and signaling molecules (Peñuelas and Munné-Bosch, 2005; Ramel et al., 2012; Rapparini et al., 2015). In *V. caruncularis*, β-carotene degradation during DS-EDS likely generates oxidative byproducts involved in stress signaling and hormonal regulation, consistent with the marked increase in ABA under severe dehydration. In *Myrothamnus flabellifolia*, β-carotene levels decline steadily throughout desiccation, reflecting its critical role in singlet oxygen quenching and dissipation of excess excitation energy (Kranner et al., 2002). In *B*. *graminifolia*, apocarotenoids derived from the β-carotene oxidation have also been implicated in desiccation tolerance (Vieira et al., 2024). Thus, β-carotenoids depletion, stress signaling, and elevated ABA levels at EDS, appear to be a distinctive feature in *V. caruncularis*.

### Contrasting carbohydrate mobilization underpins leaf maintenance versus sprouting

The accumulation of soluble sugars during drought plays a critical protective role in many plant species (Oliver et al., 2020; Dace et al., 2023). *B*. *gentianoides* and *V*. *caruncularis* exhibit contrasting carbohydrate metabolism strategies, particularly in their starch–sugar interconversion pathways under water stress, when photoassimilation is constrained. These differences reflect divergent carbon allocation strategies: *V. caruncularis* prioritizes leaf maintenance, while *B. gentianoides* directs resources toward leaf sprouting. In *V. caruncularis*, starch reserves are hydrolysed into soluble sugars that serve osmoprotective and antioxidant functions, stabilizing cellular structures and enabling rapid metabolic reactivation upon rehydration during the rainy season. The accumulation of trehalose and galactose during the DS further supports this strategy, correlating with the species’ ability to retain leaf water, endure extreme desiccation, and resume normal function upon rehydration at BRS.

Conversely, *B. gentianoides* allocates soluble sugars to starch biosynthesis in basal leaf tissues, reflecting the gradual dehydration pattern and progressive senescence by the EDS. The persistence of chlorophyll in basal tissues likely serves as a carbon reservoir that fuels resprouting at BRS rather than enabling direct recovery from dehydration. Moreover, the absence of raffinose and galactinol indicates limited synthesis of raffinose family oligosaccharides (RFOs) in *B. gentianoides*, suggesting its drought tolerance relies primarily on sucrose, myo-inositol, and pinitol. Keller and Ludlow (1993) reported that drought stress induced pinitol accumulation in *Cajanus cajan* leaves, concurrent with decreased starch and sucrose and increased SPS activity, indicating a redirection of carbon flux toward polyol biosynthesis. Accordingly, our results are consistent with previous studies showing that desiccation-tolerant species sustain sucrose turnover, induce RFO synthesis, and fine-tune osmotic balance, while desiccation-sensitive species rely on rapid carbohydrate mobilization upon rehydration (Yobi et al., 2012; Dace et al., 2023).

Elevated levels of arabinose, xylose, and rhamnose during new-leaf sprouting in *B. gentianoides* and post-dehydration recovery in *V. caruncularis* at BRS suggest that, beyond changes in non-structural sugars, active cell wall remodeling occurs following the DS. In *Arabidopsis* and maize, increased incorporation of arabinose-enriched pectins enhanced drought resistance through improved cell‐wall flexibility and water retention (Balsamo et al., 2015; Calderone et al., 2024). In *Selaginella*, elevated xylan levels are linked to drought sensitivity, likely due to reduced cell wall porosity (Plancot et al., 2019). The accumulation of arabinose-rich polymers, such as arabinans and arabinoxylans, has been linked to enhanced cell-wall flexibility in desiccation-tolerant species (Vicré et al., 2004; Wang et al., 2009; Moore et al., 2013). Loosening the cell wall matrix facilitates cellular contraction and expansion during dehydration–rehydration cycles, helping preserve water status, membrane integrity, and essential physiological functions under drought stress.

### Energetic/osmotic regulation coupled with antioxidant defense in the dry-wet cycle

The proline accumulation from the beginning to the end of the dry season (BDS–EDS) in both species underscores its dual role as an osmoprotectant and ROS scavenger. This amino acid helps to reduce water loss and stabilize cellular structures. Beyond osmotic adjustment, proline mitigates oxidative damage by stabilizing proteins and membrane lipids, enhancing ROS detoxification pathways, and maintaining the NADPH/NADP^+^ balance (Vieira et al., 2021; Carvalho et al., 2024). Notably, in *V. caruncularis*, the peak in proline at EDS coincided with increased levels of chloroplastic glutamate, suggesting coordinated regulation of glutamate-derived osmolyte synthesis under severe water stress.

Similarly, elevated phenylalanine concentrations during DS-EDS in both species underscore its importance as a precursor for phenolic antioxidants. In contrast, valine accumulation exhibited species‐specific timing: *V. caruncularis* exhibited increased valine during DS, possibly to sustain residual respiration, while *B. gentianoides* accumulated valine predominantly at EDS, coinciding with basal leaf senescence and the onset of resprouting. This divergence may reflect differences in ATP production *via* nitrogen recycling. Branched‐chain amino acids (BCAAs), such as valine, have been implicated in stress‐induced energy metabolism, serving as alternative respiratory substrates to support ATP generation (Hildebrandt, 2018). Baluku et al. (2025) further demonstrated that BCAA accumulation correlates with elevated ATP levels in senescing leaves of *Eragrostis nindensis*.

During the transition from DS-EDS, *V. caruncularis* exhibited a unique increase in 4-aminobutanoic acid (GABA), indicating its potential dual role in nitrogen remobilization and growth-arrest signaling. GABA acts as an osmoregulator and contributes to the maintenance of carbon-nitrogen balance, while also modulating growth by inhibiting cell elongation— a response that aligns with the downregulation of cell wall-related genes such as expansins and xyloglucan endotransferases (Fernández-Marín et al., 2020). In addition, GABA plays a role in oxidative stress mitigation by supplying key metabolic intermediates, particularly succinate and NAD, when the TCA cycle is impaired (Bouché and Fromm, 2004). In contrast, tryptophan— detected exclusively in *B. gentianoides*— likely fulfils dual roles, as an osmoprotectant during DS-EDS and as a nitrogen reservoir to support new leaf formation during BRS. Elevated alanine levels between BRS and RS in this species suggest its role as a readily mobilizable nitrogen source to fuel post‐stress growth. In *V. caruncularis*, the concurrent accumulation of alanine and serine, particularly at BRS, may facilitate metabolic reactivation upon rehydration. Similar accumulation patterns have been observed in *Barbacenia graminifolia* and *Craterostigma plantagineum*, where sustained alanine and serine levels after water uptake correlate with the reactivation of the photorespiratory pathway, which precedes the complete restoration of photosynthetic function (Xu et al., 2021; Vieira et al., 2022).

During the dry season, reductions in TCA cycle intermediates—citrate, malate, and succinate—observed in both *B. gentianoides* and *V. caruncularis* likely reflect enhanced photorespiratory flux and possible NADH‐mediated inhibition of key TCA enzymes (Sicher, 2015; Obata et al., 2016). Conversely, elevated oxalic acid levels during BDS (*B*. *gentianoides*) and DS (*V*. *caruncularis*) suggest a multifunctional role in drought resistance, supporting stomatal regulation, photosynthesis, osmotic balance, and antioxidant activity (Marček et al., 2019; Gouveia et al., 2020; Chen et al., 2024). In *V. caruncularis*, the accumulation of fumarate during BDS-DS may further influence stomatal control, as malate and fumarate regulate guard cell dynamics via osmotic and energetic mechanisms (Medeiros et al., 2017).

In both species, shikimic acid levels increased between BDS and DS, preceding phenylalanine accumulation during DS-EDS. In *B. gentianoides*, this metabolic shift was associated with moderate increases in cinnamic, benzoic, and chlorogenic acids. In contrast, *V. caruncularis* displayed substantially higher levels of benzoic and caffeic acids during DS, and a pronounced accumulation of vanillic acid at EDS. These enhanced levels of phenolic intermediates support activating the flavonoid biosynthetic pathway, as evidenced by the elevated quercetin and luteolin concentrations in *B. gentianoides*. Flavonoids are widely recognized for enhancing drought tolerance, primarily by strengthening antioxidant defenses and safeguarding cellular structures from oxidative damage (Sharma et al., 2019; Liu et al., 2023; Vieira, 2023). Additionally, elevated concentrations of 3-trans-caffeoylquinic acid further reinforce antioxidant protection and mitigate high-light stress (Soviguidi et al., 2022). Notably, in the resurrection species *Barbacenia purpurea*, levels of 3-trans-caffeoylquinic acid increased 25-fold in summer compared to winter, underscoring its central role in photoprotection (Suguiyama et al., 2014).

Water deficit distinctly reshaped the profiles of organic and fatty acids across dehydration stages. In *B. gentianoides*, the reciprocal pattern of increased propanoic acid and decreased valine from BDS to DS supports a metabolic reallocation favoring flavonoid biosynthesis at EDS. Li et al. (2024) proposed that propanoic acid enhances drought resistance by promoting energy metabolism and antioxidant defense, partly through the regulation of branched‐chain amino acid degradation and the upregulation of flavonoid biosynthesis. Moreover, both species showed elevated levels of long‐chain fatty acids—stearic, palmitic, and oleic—particularly from EDS through BRS. These compounds likely contribute to membrane stabilization, mitigate mechanical damage, and buffer against temperature extremes (Vieira et al., 2022). Concurrently, the build-up of diverse organic acids may help reduce water loss, extend energy availability during prolonged drought, and provide key metabolic precursors for maintaining ROS homeostasis.

### Differential phytohormonal responses drive metabolic adjustments and leaf senescence during progressive water deficit

Phytohormonal signaling pathways are well-known as key mediators of drought resistance and desiccation tolerance (Djilianov et al., 2013; Ullah et al., 2018; Chhaya et al., 2021; Salvi et al., 2021). In *B. gentianoides* and *V. caruncularis*, ABA, JA, and SA levels increased progressively throughout the dry season, peaking during the DS. The early rise in ABA and JA at the BDS and DS appears crucial for conserving water and mitigating further dehydration, primarily through the induction of stomatal closure and the stimulation of osmolyte production. This hormonal response corresponds with the accumulation of trehalose, proline, xylitol, galactinol, and myo-inositol in both species. In *V. caruncularis*, the sustained ABA accumulation at EDS likely represents a response to extreme leaf dehydration, potentially triggering dehydration-responsive genes that help stabilize cellular structures and proteins under severe water deficit. A similar late-stage ABA response has been documented in *Sporobolus stapfianus*, where this hormone plays a key role in inducing protective proteins such as LEAs and HSPs (Yobi et al., 2017).

Harb et al. (2010) proposed that plants establish a new homeostasis during the late stages of drought through the alteration in jasmonate-ABA balance, resulting in reduced growth. In *V. caruncularis*, the stable JA levels observed during DS and EDS likely reflect a growth-suppressive acclimation response to prolonged drought conditions. In contrast, *B. gentianoides* declined JA levels at EDS, suggesting a distinct regulatory strategy. In both species, the accumulation of secondary metabolites (such as cinnamic, coumaric, shikimic, caffeic acids, and quercetin) and aromatic amino acids (phenylalanine and tryptophan) appears to be associated with JA signaling, as many steps in their biosynthesis are known to be JA-inducible (Jogawat et al., 2021).

A key distinction between resurrection and non-resurrection plants is their ability to avoid drought-induced senescence (Blomstedt et al., 2018). In *B. gentianoides*, elevated levels of the ethylene precursor ACC— known to promote programmed cell death and senescence— were detected exclusively at EDS, coinciding with leaf senescence and subsequent resprouting during the RS. This stress-induced senescence is characterized by chloroplast dismantling and protein degradation, as reflected by decreased chlorophyll content and elevated amino acid levels at EDS. Additionally, increased ROS levels may further contribute to ethylene-mediated leaf senescence in this species. In contrast, the absence of ACC accumulation in *V. caruncularis* suggests a capacity to suppress dehydration-triggered senescence, enabling rapid photosynthetic recovery following rehydration. Similarly, non-senescent leaves of the resurrection species *Borya constricta* turn yellow yet survive dehydration, while senescent leaves fail to recover (Griffiths et al., 2014). Likewise, ABA-induced leaf yellowing in *V. caruncularis* is associated with desiccation tolerance rather than senescence.

## Conclusions

**Figure 9:**
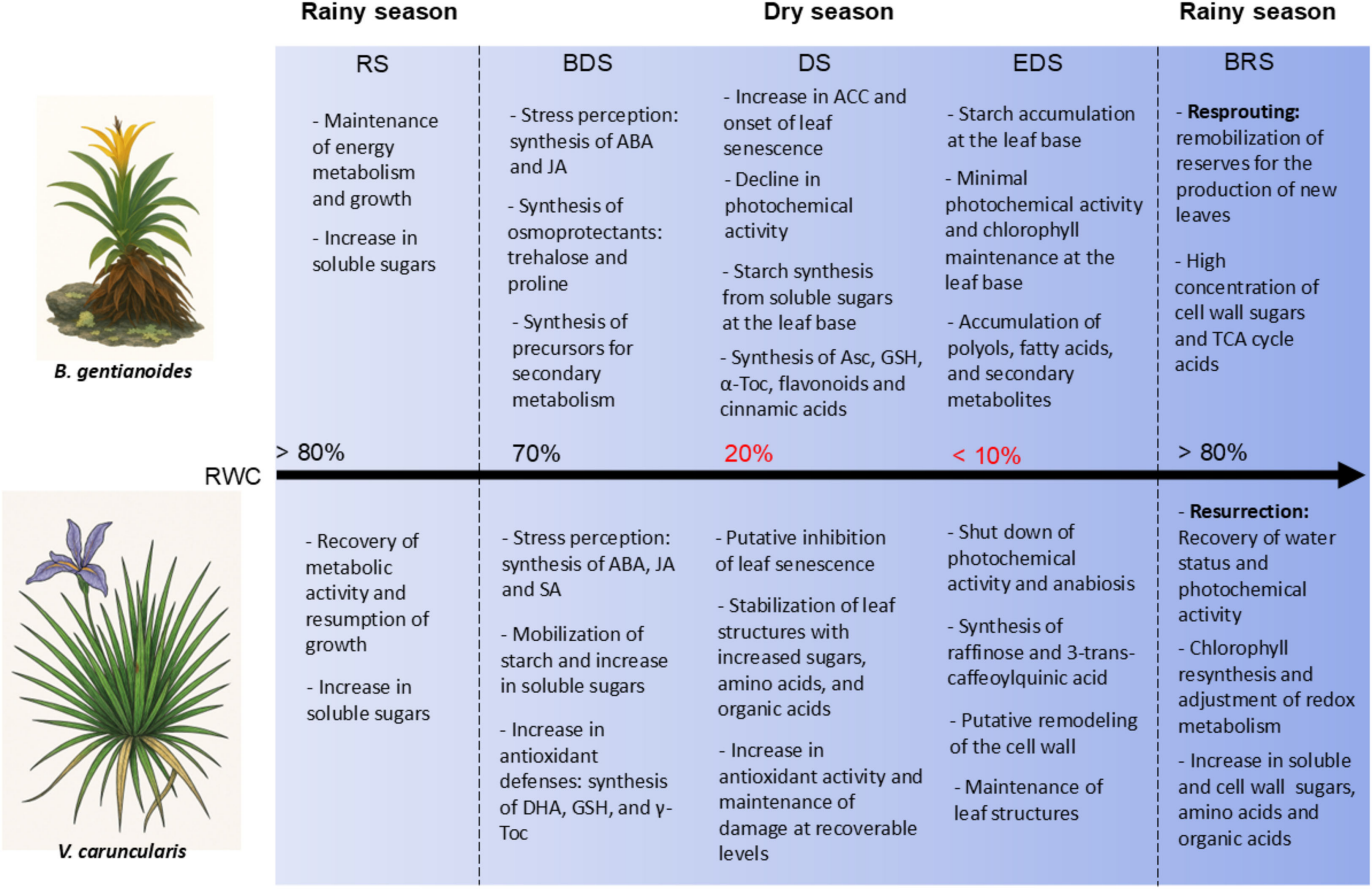
The schematic model summarizing physiological and metabolic responses to drought and desiccation in the co-occurring species *B*. *gentianoides* and *V*. *caruncularis* growing under field conditions during the seasonal cycle. RS, rainy season; BDS, beginning of the dry season; DS, dry season; EDS, end of the dry season; BRS, beginning of the rainy season.

*Barbacenia gentianoides* and *Vellozia caruncularis*, two species that coexist under natural conditions, survive seasonal water scarcity in the *campos rupestres* by employing distinct drought resistance and desiccation tolerance strategies. While *V. caruncularis* suppresses drought-induced senescence in younger leaves, *B. gentianoides* invests in resprouting following senescence-driven carbon reallocation from the pre-existing leaves. These contrasting strategies are underpinned by physiological traits and key metabolic shifts in carbohydrate, amino acid, and secondary metabolite pathways underscore these differences. *V. caruncularis* prioritizes osmoprotection and ROS scavenging through starch degradation, raffinose and trehalose accumulation, and antioxidant synthesis. In contrast, *B. gentianoides* redirects carbon resources toward polyol synthesis and starch storage in leaf bases, favoring regeneration over immediate recovery. Differences in flavonoid and phenylpropanoid metabolism and phytohormonal regulation further highlight species-specific responses. *V. caruncularis* sustains the accumulation of 3-trans-caffeoylquinic acid and exhibits higher ABA and JA levels to modulate responses to extreme dehydration, whereas *B. gentianoides* incorporates ethylene-mediated senescence as part of its survival strategy (**Figure 9**).

Considering that *campos rupestres* exhibit pronounced topographic and edaphic gradients that strongly influence the distribution of Velloziaceae species (Alcantara et al., 2015), *V. caruncularis* colonizes microhabitats with sandy, shallow, and nutrient-poor soils, where soil moisture is scarce. This slow-growing species retains desiccated leaves and relies on specialized physiological and metabolic traits to withstand harsh abiotic conditions. In contrast, *B*. *gentianoides* establishes in rock crevices that may be deeper, moister, and capable of retaining organic matter. In this case, the microhabitat enables the species to reallocate foliar resources during the dry season and use them for resprouting in the rainy season. Studies by Blignaut and Milton (2005) and Rafiee et al. (2023) demonstrate that, as resource availability increases, competition intensifies, often excluding desiccation-tolerant species. Although they occur simultaneously, their survival strategies suggest that competition in rocky outcrops likely confines *V*. *caruncularis* to the most stressful habitats, where abiotic constraints limit the success of more competitive species such as *B*. *gentianoides*.

In water-limited environments, such as the *campos rupestres*, distinct water-use strategies and unique physiological, morphological, and metabolic traits play a crucial role in reduce competition for scarce water (Jactel et al., 2009). Identifying these strategies is essential for understanding their role in shaping community dynamics (Suding et al., 2008). Such knowledge is also crucial for predicting vegetation patterns, assessing ecosystem functioning, and guiding conservation efforts in the face of climate change. However, the responses of native species to drought and desiccation under natural conditions remain poorly explored, particularly in rock outcrop environments. From this perspective, our findings contribute to a deeper understanding of the metabolic signatures of Velloziaceae species in these ecosystems.

Future studies involving this pair of species would benefit from broader approaches, including multi-site and multi-year sampling, as well as the integration of physiological and metabolic analyses with transcriptomic and genomic data. These strategies would enhance our understanding of the molecular mechanisms underlying drought responses in these species. Additionally, our study focused exclusively on aboveground leaf tissues, without assessing root responses. However, it is reasonable to assume that the belowground systems play a significant role in the physiological drought responses of *B*. *gentianoides* and *V*. *caruncularis*, reflecting their divergent strategies for coping with water scarcity. Therefore, to achieve a more comprehensive understanding of these adaptive strategies, it is essential to adopt a whole-plant perspective and extend research efforts to include other co-occurring species in the *campos rupestres* that exhibit contrasting mechanisms for coping with water limitation.

## Data Availability

The data that support the findings of this study are available from the corresponding author upon reasonable request.
